# Comparison of temperature measurements of pacemaker leads in a 1.0T high field open MRI and a 1.5T classic cylindrical MRI: initial results

**DOI:** 10.1186/1532-429X-13-S1-P64

**Published:** 2011-02-02

**Authors:** Sebastian A Seitz, Julian J Ebner, Gerriet Petry, Evangelos Giannitsis, Olaf Dössel, Hugo A Katus, Henning Steen

**Affiliations:** 1 Karlsruhe Institute of Technology (KIT), Karlsruhe, Germany; 2 University Heidelberg, Heidelberg, Germany; 3 Heidelberg Health System, Heidelberg, Germany

## Introduction

As of today, the use of MRI procedures on patients with implanted cardiac pacemakers is prohibited due to safety issues. The implants can interact with the RF fields of the MRI device. The most hazardous effect is heating at the tip of the lead, less dangerous are sensing errors and malfunctions of the devices, because they disappear completely after the procedure. The majority of the previous studies used classic cylindrical whole-body MRI systems. The influence of different alignments of the pacemaker/lead system and the RF fields were evaluated by comparing temperature changes occurring in a cylindrical device with the effects induced in a high field open MRI (HFO) system.

## Purpose

Compare the influence of different RF field properties in cylindrical and open MRI systems in terms of induced heating in the vicinity of a pacemaker/lead system.

## Methods

Two high energy MR-sequences with artificial ECG-triggering at 60/min were used on the 1.0T HFO and the cylindrical system: 1. T2-TSE (TR/TE=177/38ms;TSE-factor=16; time=58s;flip-angle=90°); 2. 3-D bTFE (TR/TE=4.7/2.4ms;TFE-factor=6;time=382s;flip-angle=70°;TFE-shot-duration=34ms;TFE-shots=532). A conventional bipolar cardiac pacing lead (Medtronic Capsurefix Novus) was connected to a St. Jude Frontier II pacemaker. It was placed on a Plexiglas plate in a saline-filled Plexiglas phantom. Two configurations were tested in both systems, one with the supporting plate of pacemaker and lead flat and one in a 90° tilted configuration imitating the orientation in the regular and the opposite MRI system. Temperature measurements were captured with a fiber-optic measurement system.

## Results

**Figure 1:**
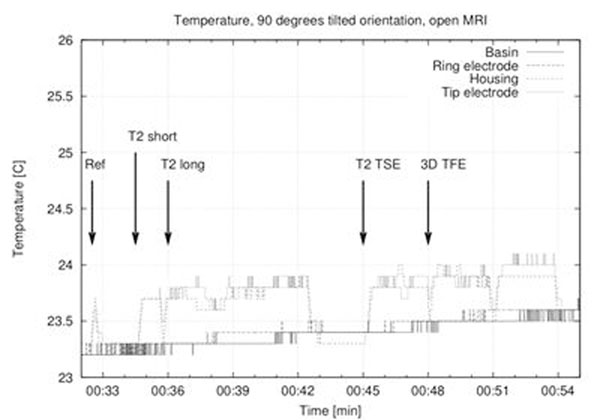
Temperature at four different spots while exposed to a series of MRI sequences in the open MRI system (90 degrees tilted).

**Figure 2:**
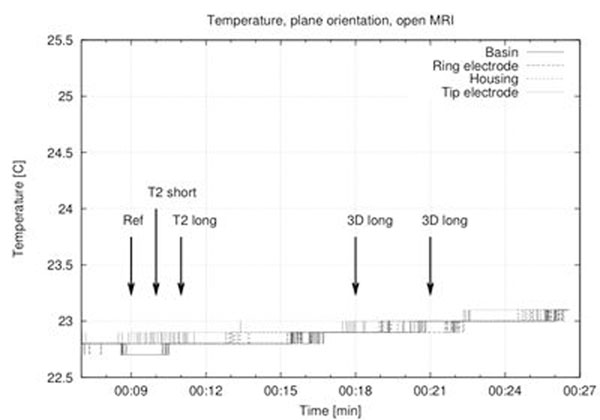
Temperature at four different spots while exposed to a series of MRI sequences in the open MRI system (flat orientation).

Highest temperature rise measured in the cylindrical system was 1.8 °C with the supporting plane in a position that mimics the normal pacemaker orientation. The highest temperature increase in the open MRI (0.6 °C) was observed in an upright configuration (see fig. 1). In the regular position, nearly no heating was induced in the lead (see fig. 2).

## Conclusions

The comparative in-vitro measurements showed a significant influence of the RF field orientation. By tilting the pacemaker/lead system, effects observed in the cylindrical MRI system could be reproduced in the open MRI device. This study could confirm the advantageous properties of the open MRI system for patients with implanted cardiac pacemakers.

